# The major inducible small heat shock protein HSP20-3 in the tardigrade *Ramazzottius varieornatus* forms filament-like structures and is an active chaperone

**DOI:** 10.1016/j.cstres.2023.12.001

**Published:** 2023-12-05

**Authors:** Mohammad Al-Ansari, Taylor Fitzsimons, Wenbin Wei, Martin W. Goldberg, Takekazu Kunieda, Roy A. Quinlan

**Affiliations:** a Department of Biosciences, Upper Mountjoy Science Site, University of Durham, Durham DH1 3LE, UK; b Department of Biochemistry, Health Sciences Centre, Kuwait University, Kuwait; c Department of Biological Sciences, The University of Tokyo, Japan; d Department of Biological Structure, University of Washington, Seattle, WA 98195, USA

**Keywords:** Chaperone, Small heat shock protein, chaperone oligomerisation, Filament-like, Protein condensation, Cryptobiosis, Tardigrade

## Abstract

The tardigrade *Ramazzottius varieornatus* has remarkable resilience to a range of environmental stresses. In this study, we have characterised two members of the small heat shock protein (sHSP) family in *R. varieornatus*, HSP20–3 and HSP20–6. These are the most highly upregulated sHSPs in response to a 24 h heat shock at 35 ^0^C of adult tardigrades with HSP20–3 being one of the most highly upregulated gene in the whole transcriptome. Both *R. varieornatus* sHSPs and the human sHSP, CRYAB (HSPB5), were produced recombinantly for comparative structure-function studies. HSP20–3 exhibited a superior chaperone activity than human CRYAB in a heat-induced protein aggregation assay. Both tardigrade sHSPs also formed larger oligomers than CRYAB as assessed by size exclusion chromatography and transmission electron microscopy of negatively stained samples. Whilst both HSP20–3 and HSP20–6 formed particles that were variable in size and larger than the particles formed by CRYAB, only HSP20–3 formed filament-like structures. The particles and filament-like structures formed by HSP20–3 appear inter-related as the filament-like structures often had particles located at their ends. Sequence analyses identified two unique features; an insertion in the middle region of the N-terminal domain (NTD) and preceding the critical-sequence identified in CRYAB, as well as a repeated QNTN-motif located in the C-terminal domain of HSP20–3. The NTD insertion is expected to affect protein-protein interactions and subunit oligomerisation. Removal of the repeated QNTN-motif abolished HSP20–3 chaperone activity and also affected the assembly of the filament-like structures. We discuss the potential contribution of HSP20–3 to protein condensate formation.

## Highlights

• Characterisation of the two most highly induced small heat shock proteins (sHSPs), HSP20-3 and HSP20-6 after a 24 h heat shock in the tardigrade, *Ramazzottius varieornatus*.
• HSP20-3 as the first sHSP that naturally forms filament-like structures as well as other oligomeric structures.
• The oligomeric particles and filament structures appear inter-related.
• Chaperone activity evidenced for both HSP20-3 and HSP20-6 in comparison with CRYAB using the heat aggregation of the client protein, malate dehydrogenase.
• A repeated QNTN-motif in the C-terminal domain of HSP20-3 was identified to be responsible for its *in vitro* chaperone activity.

## Graphical Abstract

**Figure:**
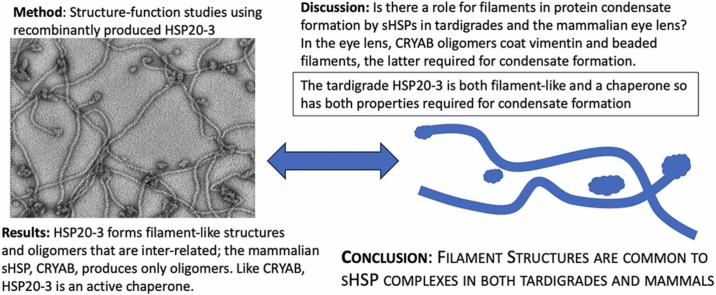

## Introduction

Within the animal kingdom, tardigrades are known to have exceptional stress resistance capabilities^1^, ^2^. One such tardigrade is *Ramazzottius varieornatus,* a member of the *Eutardigrada* class within the *Tardigrada* phylum^3^. A complete genome is available^4^, which revealed the expansion of some gene families involved in stress resilience compared to other metazoans, e.g., Superoxide dismutase and MRE11^4^. Other pathways, such as the stress response to hypoxia (HIF1a, PHD and VHL), to oxidative (TSC1/2) and to genotoxic stress (REDD1;^5^) were lost. There was, however, an expansion of tardigrade-specific genes encoding heat-soluble proteins^6^. These include the cytoplasmic abundant heat soluble (CAHS), mitochondria abundant heat soluble protein (MAHS) and secretory abundant heat soluble (SAHS) proteins that were found in a broad spectrum of species belonging to the Eutardigrade class^4^, ^7^, ^8^. Many of these heat-soluble proteins, especially the CAHS proteins, are characteristically intrinsically disordered^9^, ^10^ and they are very important to the remarkable resistance to extreme stresses, such as desiccation^11^, ^12^. They can stabilise other proteins^12^, ^13^, ^14^, form biomolecular condensates^6^, ^14^ and help regulate the residual water environment^15^, ^16^. Indeed, regulating the egress and ingress of water and solutes^16^ as well as the prevention of protein aggregation and precipitation is key to the success of *R. varieornatus* and other *Eutardigrada* members to surviving the extreme stress experienced during freezing, dehydration and rehydration as the animal enters and exits the ametabolic state called the tun^1^, ^16^. This remarkable physiological condition is called anhydrobiosis and it is one of several cryptobiotic adaptions found in *R. varieornatus* and other tardigrades^1^. In the tun state, the animal becomes a third shorter along its long axis^17^. This is accompanied by a reduction in both cell and mitochondrial size^17^ and most noticeably by the appearance of two types of secretory active cells, one with a large number of vesicles and the other rough endoplasmic reticulum^18^, ^19^ as water and solutes levels are regulated^16^, ^20^. Comparison of the molecular adaptions across *Eutardigrada* (*Hypsibius exemplaris, R. varieornatus*, *Richtersius coronifer*) and *Heterotardigrada*^21^ with two other related invertebrates, the arthropod, *Drosophila melanogaster* and the nematode, *Caenorhabditis elegans* and various vertebrates and yeast^7^ established that proteins in addition to the tardigrade specific proteins (CAHS, MAHS, SAHS) are necessary for the unique molecular adaptions that supports cryptogenic responses to extreme stresses^7^, ^21^. With this in mind, we investigated the small heat shock protein (sHSP) family present in *R. varieornatus* because sHSPs have properties in common with CAHS and SAHS proteins, such as their ability to prevent protein aggregation and precipitation^22^, ^23^, ^24^, ^25^, form biomolecular condensates^26^, ^27^, ^28^ and to be upregulated in response to cold and heat shock of *R. varieornatus*^29^, ^30^.

In *R. varieornatus* there are eight sHSPs identified by the presence of an α-crystallin domain (ACD; Pfam entry PF00011; PROSITE entry PS01031). All but one in this protein family have both N- and C-terminal domains that are predicted to be unstructured. The one exception is the sHSP-domain containing protein (Gene RvY_18177–1), which lacks both the unstructured C-terminal domain (CTD) and the C-terminal end of the ACD^31^, ^32^. We have tentatively assigned this as HSP20–8, but structure-function studies are needed to confirm its sHSP credentials. The predicted sequence for HSP20–8 is just 95 residues and contains an incomplete sHSP domain that would be expected to disrupt the IPI-β4/β8 surface interaction that is key to the oligomerisation of CRYAB (HSPB5;^33^, ^34^), but these features are present in the other seven sHSPs in *R. varieornatus*. The N- and C-terminal unstructured domains of sHSPs also regulate the interaction with client proteins as well as sHSP subunit oligomerisation^35^, ^36^, ^37^, ^38^, ^39^.

Using publicly available expression datasets^21^, ^28^, ^29^, ^40^ we have characterised the sHSP expression profile in response to a 35 ^0^C heat shock for *R. varieornatus*. A previous transcriptomic study had identified that four sHSPs were upregulated,^29^, ^30^, but their relative upregulation as well as their structural-functional capabilities were not characterised. We identify HSP20–3 and HSP20–6 as the two most highly upregulated sHSP genes after heat shock at 35 ^0^C. It transpires that the HSP20–3 gene is the most highly upregulated within the transcriptome of heat shocked *R. varieornatus* (29, 30; Nadja Møbjerg, personal communication). Neither sHSP was upregulated upon cold shock and each showed distinctive developmental expression patterns with HSP20–6 being upregulated in the tun state, but not HSP20–3. We produced both HSP20–3 and HSP20–6 recombinantly by expressing each in *E.coli* so that for the first time the structure-function properties of both proteins could be investigated for this tardigrade and compared to those data obtained for the two sHSPs, HSP24.6 and HSP21 from *Hypsibius exemplaris*^41^. Here we report that both HSP20–3 and HSP20–6 were found to be active chaperones as compared to human CRYAB (HSPB5). We established that the C-terminal repeated QNTN-motif present in HSP20–3 is very important to its chaperone activity. Whilst HSP20–6 formed oligomeric particles similar to those formed by CRYAB, HSP20–3 formed filament-like structures as well as particles indicating a different assembly profile. This filament-like form was not reported for either of the two *Hypsibius exemplaris* sHSPs^41^, but we note that CAHS proteins can also form filament-like structures^14^, as can CRYAB under particular heat and denaturing conditions^42^ and in complex with some client proteins^43^. We present evidence to show that these two polymeric forms reported here for HSP20–3 are interrelated and we also show that the C-terminal repeated QNTN-motif is not solely responsible for this polymeric plasticity, but that its removal severely compromises its chaperone activity.

## Materials and methods

### Cloning, expression and purification of HSP20-3, HSP20-6, HSP20-3NCT and CRYAB

The small heat shock proteins were cloned from the YOKUZUNA-1 strain of *R. varieornatus* and inserted into the vector pME18sf3 at the DRAIII site. The coding sequence was then PCR amplified adding a *Nde*I site to the 5′ end and either a *Eco*RI or *Bam*HI site to the 3′ end of the coding sequence. This facilitated subcloning into the selected bacterial expression vector, based upon the pET system^44^. After DNA sequencing the coding sequences were confirmed. The primers used to subclone HSP20–3 and HSP20–6 are detailed in Table 1 as too are the primers used to generate the mutant HSP20–3NCT, where the CTD has been truncated to remove the repeated QNTN-motif sequences.

**Table 1:** Primer sequences used to PCR amplify coding sequences from RvY_03967 (HSP20–3, HSP20–3NCT) and RvY_08665 (HSP20–6).

*R.varieornatus* sHSP	Primer Sequences
HSP20–3	Forward Primer (*Nde*I site) 5′ CATATGAGCATGCAACGCTACGACGACTACAACGATTACGGCAATCGCCAAAT GCGGCCTCATCGCG 3′ Reverse Primer (EcoR1 site) 5′ GAATTCATTTGTTCTGGTTGGTGTTCTGGTTGGTGTTCTGGTTGGTGTTCTGGTTGG TGTTCTGGTTGGTGTTCTGGCTAC 3′
HSP20–3NCT	Reverse primer to truncate HSP20–3 at G191 (*Eco*RI site) 5′ GAATTCTCAGCCAGAGTTCTGAATGGAGTTC 3′
HSP20–6	Forward primer (*Nde*I site) 5′CATATGTCGCGGAATCTAGCTCGTCTGC 3′ Reverse primer (*Hin*dIII site) 5′ AAGCTTATTCGTGCTTGATGGGGATGTTATGC 3′

Expression constructs were generated by PCR and, after sequencing, were sub-cloned into pET23b vectors. A stop codon preceded the C-terminal hexa-histidine tag, so affinity purification using this tag was not possible. The expression constructs were transfected into *E.coli* BL21 (pLysS), and the protein products induced by the addition of 1 mM IPTG to the culture once the OD_600_nm reached 0.6. After 3 h, the bacteria were harvested by centrifugation and soluble protein extracts prepared as described previously for CRYAB^45^. Recombinant proteins were purified by ion exchange chromatography using a Q-Sepharose fast flow column gradient Tris EDTA Buffer (20 mM Tris-HCl, 20 mM NaCl, 1 mM DDT, 1 mM EDTA). A Superose 6 10/300 column (Cytiva Lifesciences) was used as a final purification step and the proteins were eluted in 0.5 M NaH_2_PO_4_, 0.5 M Na_2_HPO_4_, 150 mM NaCl, 5 mM EDTA pH8.0. A protein standard mix (Sigma-Aldrich, Poole, UK) comprising thyroglobulin bovine (670,000 Da), bovine y-Globulin (150,000 Da), chicken egg albumin (44,300 Da), ribonuclease A (13,700 Da), p-Aminobenzoic acid (137 Da) was used to calibrate the column. Fractions containing the protein of interest were pooled and concentrated in an Amicon Stirred Cell and ultracell regenerated cellulose membranes with 10 kDa cut-off (Merckmillipore, Watford, UK). Recombinant human CRYAB was also produced recombinantly in *E.coli* as described previously^46^. Protein products were quality controlled by SDS-PAGE, followed by mass spectrometry.

### SDS-PAGE and mass spectrometric analyses

Protein samples were resuspended in SDS sample buffer (1 mM EDTA pH 7.8, 50 mM Tris-HCl pH 6.8% and 1% (w/v) SDS) and then mixed with Laemmli sample buffer^47^. In some cases proteins were precipitated using a methanol-chloroform method^48^ to remove salt that could affect the observed electrophoretic mobility. Protein samples in SDS-PAGE sample buffer were separated on NuPAGE™ 4–12% (w/v) acrylamide gradient gels using a Bis-Tris buffer system (ThermoFisher Scientific, Warrington, UK). Protein standards (PageRuler, 10–180 kDa; ThermoFisher Scientific, Warrington, UK) were included. Gels were stained with Coomassie brilliant blue R-250 (0.25% (w/v); Merckmillipore, Watford, UK) to detect the separated proteins, de-stained using methanol and acetic acid solutions before being imaged using a Thermofisher Scientific iBRIGHT imaging system.

Purified protein identity was confirmed by proteomic analysis of bands excised after SDS-PAGE (LC-MS analysis) as described previously^49^. Briefly, trypsin was added to the dried, excised band and digestion was performed overnight at 37 ^0^C. Digestion was stopped by adding trifluoroacetic acid (TFA) and the eluted peptides were cleaned using StageTips as described^50^. Peptide analysis was performed on a SCIEX TripleTOF 6600 mass spectrometer linked to an Eksigent nanoLC 425 chromatography system via a 50-micron ESI electrode in a DuoSpray source (SCIEX). Identified peptides were then mapped onto the primary sequence for HSP20–3, HSP20–6, HSP20–3NCT and CRYAB (Supplementary Fig. 1) to confirm the identity of the purified proteins.

### Transmission electron microscopy and negative staining of protein samples

Recombinant proteins were diluted in 10 mM Tris-HCl pH 8.0, 5 mM EDTA, 50 mM NaCl to approximately 25 μg/ml. The Valentine method was used to negatively stain samples spread onto carbon films^51^ with 1% (w/v) uranyl acetate (Agar Scientific, Stanstead, United Kingdom). Samples were spread on 400 mesh copper grids (EM Resolutions Ltd, Sheffield, UK) and imaged in a Hitachi H-7600 transmission electron microscope (TEM, Hitachi High-Technologies, Tokyo, Japan) at an accelerating voltage of 100 kV. Images were acquired using Radius software v2.1 with a Xarosa digital camera (EMSIS GmbH, Münster, Germany) and montages made using Adobe Photoshop (v24.7.0; Adobe Systems, San Jose, CA).

### Bioinformatic Analyses

The RNA-seq data sets for *R. varieornatus* that included the small heat shock protein expression after heat shock^29^, ^30^, cold shock^40^ and the different life stages^21^ were downloaded from the European Nucleotide Archive^52^ at EMBL-EBI under accession number PRJEB49649 for heat shock and PRJEB47628 for cold shock, PRJNA369262 and PRJNA533981 for the different life-cycle stages and states including the tun as the anhydrobiotic stage^53^. *Ramazzottius varieornatus* reference genome Rvar_4.0 data set^54^, including the genome sequences and general feature format file, was downloaded from the NCBI genome database^54^. Sequence reads were processed using fastp (v0.23.4;^55^) to remove low quality reads with the default settings and aligned to the Rvar_4.0 genome using STAR (v 2.7.11a;^56^) to obtain read count per gene. The eight small HSPs (HSP20–1, HSP20–2, HSP20–3, HSP20–4, HSP20–5, HSP20–6, HSP20–7 and HSP20–8) did not have alternatively spliced coding sequences. Read count per gene was analysed using DESeq2 v 1.40.2^57^ to get p values and log2 fold change of all genes including the eight *R. varieornatus* sHSP genes. DESeq2 normalised counts of the eight small HSP genes were plotted using ggplot2 (v3.4.3;^58^, ^59^).

Sequence alignment of human CRYAB (P02511; CRYAB_Human), *R. varieornatus* HSP20–3 (A0A1D1UZ79; A0A1D1UZ79_RAMVA) and HSP20–6 (A0A1D1VFU2; A0A1D1VFU2_RAMVA) using sequences downloaded from UniProt^60^ was produced using COBALT^61^. NCBI Tree Viewer (Treeviewer JS version: 1.19.4) was used to plot phylogenic tree to identify sHSP homologues between *R. varieornatus* and *Hypsibius exemplaris*. AlphaFold2^62^ was used to predict secondary sequence features for HSP20–3^63^, HSP20–6^64^ and HSP20–8^32^.

### Chaperone client protein assays

Malate Dehydrogenase (MDH) (Sigma-Aldrich, UK) was dialysed for 16 h at 4 °C against 50 mM Tris-HCl, 2 mM EDTA, pH8.0 and diluted to 0.89 mg/ml. The assay mixture in a volume of 220 µl contained MDH and *R. varieornatus* HSP20–3, HSP20–6 and HSP20–3NCT in a 4:1 ratio (client: chaperone). Recombinant human CRYAB was used as a chaperone control, added in the same 4:1 ratio as for the tardigrade proteins. Time-dependent light scattering was measured at 350 nm every 30 s over 60 min at 45 °C using a Beckman DU640 spectrophotometer equipped with a 6-place cuvette and a Peltier heating controller^65^, ^66^. Experiments were repeated 5–6 times. To test for statistical significance (p value <0.5), a one tailed T-test for datasets of equal variance for protein pairs was used. The optical density value at 60 min was corrected for its starting value to give a final OD_350_ difference at the conclusion of the assay.

## Results

### R. varieornatus HSP20-3 and HSP20-6 are the most highly upregulated sHSP genes after heat shock and during development and tun formation

**Fig. 1:**
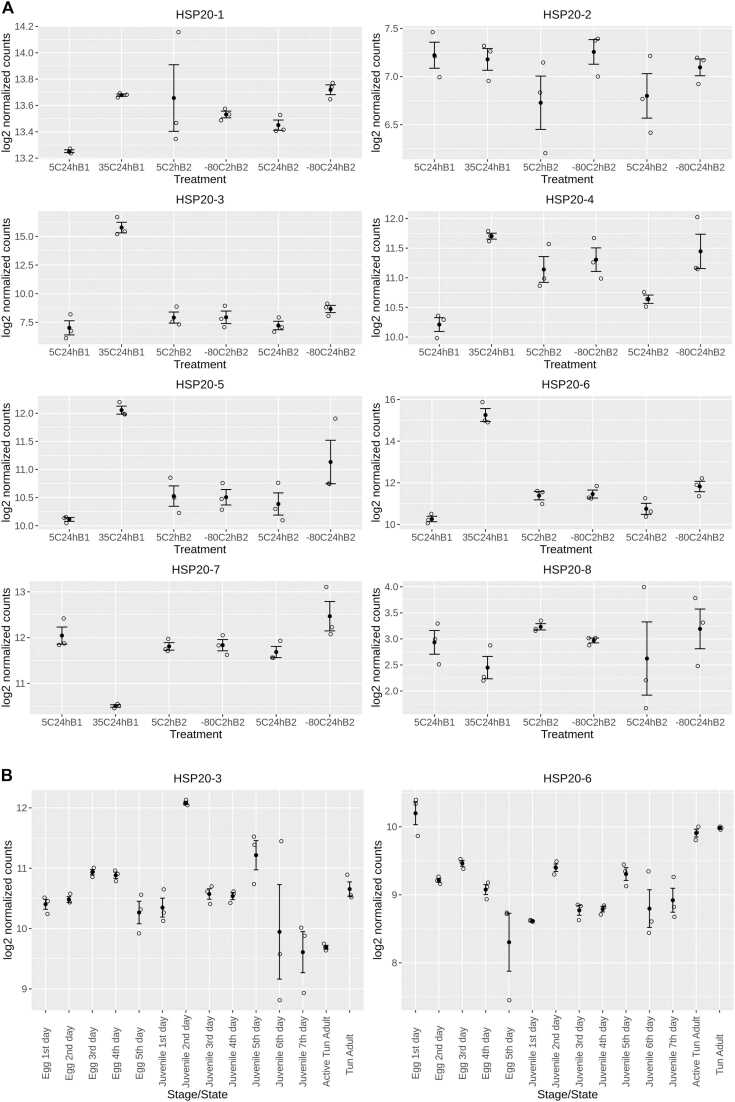
Expression (log2 normalised counts) of small heat shock genes in R.varieornatus. Each group has three replicates (white circles). The mean values (black dots) and their corresponding ± standard error are shown (solid capped lines). (A) The expression of the eight sHSP genes in the 18 samples available under the ENA accession numbers PRJEB49649 and PRJEB47628. Sample groups were labelled according to treatment temperature (5^0^C, 35^0^C, −80^0^C), time (2 h, 24 h) and data set (B1 for PRJEB49649, B2 for PRJEB47628). After a 24 h heat shock at 35 ^0^C (labelled as 35C24hB1), both HSP20–3 and HSP20–6 showed a dramatic upregulation in their gene expression compared to the other sHSP genes (see also Table 2). (B) The expression of HSP20–3 and HSP20–6 of 42 samples at different developmental stages and states available under the ENA accession numbers, PRJNA369262 and PRJNA533981. Expression of HSP20–3 is elevated in the 2 day old juvenile sample, whilst HSP20–6 is elevated in the 1 day old egg and in the tun state. The full dataset for the expression of sHSPs during development can be found in Supplementary Fig. 2.

Previous studies on the heat shock response of adult *R. varieornatus* identified four small heat shock proteins (sHSP) upregulated after a 35 ^0^C heat shock for 24 h^29^, ^30^. We have now analysed these data and confirmed that the gene expression of four small heat shock proteins (HSP20–3, HSP20–4, HSP20–5 and HSP20–6) were significantly upregulated after heat shock with p values < 1 E-20 and log 2 fold change > 1 (Table 2 and Fig. 1A). HSP20–3 and HSP20–6 had the largest and second largest log2 fold changes of 8.62 and 5.03 respectively. Amongst the genes significantly (false discovery rate adjusted p value < 0.05) upregulated by heat shock, HSP20–3 is ranked the 3rd in terms of log2 fold change. The change in expression of HSP20–3 and HSP20–6 in response to extreme cold temperatures (−80^0^ C) was by comparison dwarfed by the response of these genes to a 24 h heat shock at 35 ^0^C. Investigating how the expression levels of HSP20–3 and HSP20–6 changed during development and in the tun stage (Fig. 1B) showed that HSP20–6 was elevated both in the one day egg and tun stage, whilst HSP20–3 showed elevated expression in the 2 day old juvenile tardigrade (Fig. 1B).

**Table 2:** Log2 fold change and p-value in expression of sHSP genes in R. varieornatus after treatment at 35 ^0^C for 24 h compared with those at 5 ^0^C for 24 h.

UniprotKB Identifier	Gene ID	Protein Name	NCBI/ENA Identifier	p value	log2 Fold Change
A0A1D1UF75	RvY_01018	HSP20–1	GAU88286.1	3.05E-09	0.41
A0A1D1UIJ9	RvY_01054	HSP20–2	GAU88340.1	0.74	-0.07
A0A1D1UZ79	RvY_03967	HSP20–3	GAU91773.1	1.27E-26	8.62
A0A1D1VW57	RvY_15789	HSP20–4	GAV05697.1	1.20E-30	1.46
A0A1D1W9I0	RvY_17905	HSP20–5	GAV08174.1	1.53E-77	1.93
A0A1D1VFU2	RvY_08665	HSP20–6	GAU97348.1	5.27E-103	5.03
A0A1D1VPU4	RvY_13475	HSP20–7	GAV02981.1	2.73E-23	-1.58
A0A1D1W4T5	RvY_18177	HSP20–8	GAV08495.1	0.50	-0.50

### Secondary structure prediction for HSP20-3 and HSP20-6

**Fig. 2:**
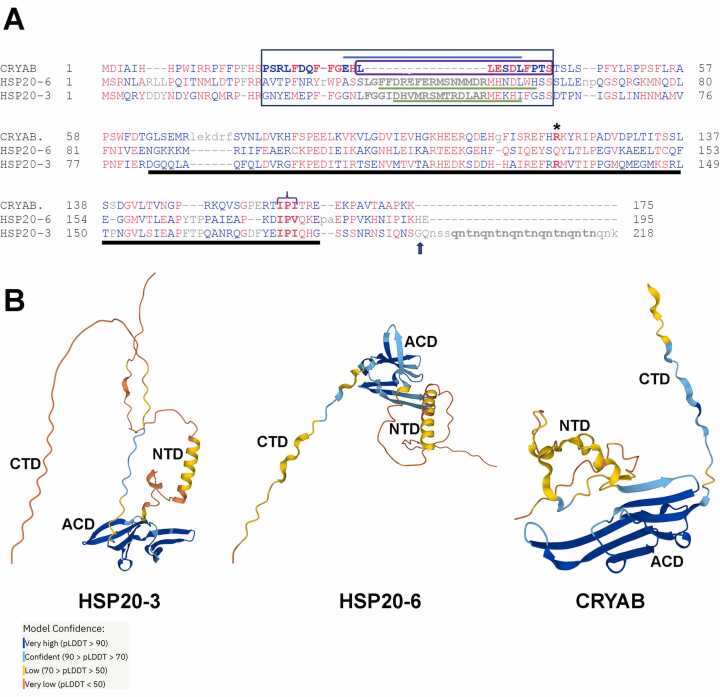
Sequence alignment of HSP20–3 and HSP20–6 compared to human CRYAB. (A) The COBALT alignment shows highly (red) and less (blue) conserved residues. Regions lacking conservation (grey) are also indicated. The ACD domain (residues 64–164 in CRYAB) is underlined and the middle-sequence of the NTD^39^ is boxed. The critical-sequence within this region (purple rectangle) overlaps with a β-sheet (blue line) within the CRYAB NTD, but in HSP20–3 and HSP20–6 there is a 17-residue insertion (grey lowercase) that overlaps with an AlphaFold2 predicted α-helix (green line) in both cases. Within the ACD, the R120 residue (asterisk) and IPI motif (purple bracket) are indicted. The arrow indicates G191 in the HSP20–3 sequence and the insertion point for a stop codon to construct HSP20–3NCT and remove the QNTN-repeat (bold, lowercase) in the CTD. (B) AlphaFold2 prediction for secondary structure features of HSP20–3^63^ and HSP20–6^64^. The NTD, ACD and CTD domains are indicated for HSP20–3, HSP20–6 and CRYAB. A low confidence α-helix is predicted in HSP20–3 and HSP20–6 within the NTD and corresponds to the middle-sequence of the NTD^39^.

New sHSP protein members are identified via the corresponding PROSITE ACD and this was also the case for identifying members of the sHSP protein family in the *R. varieornatus* genome^4^. We have concentrated on HSP20–3 and HSP20–6 given their importance to the heat shock response of this tardigrade and show the alignment of their sequences to human CRYAB (Fig. 2A). Sequence conservation in the NTD, ACD (Fig. 2A, black line) and CTD are indicated, although sequence identity per se is low. Within the NTD, we were intrigued by the middle sequence (Fig. 2A, black rectangle) and its “critical-sequence” (Fig. 2A, purple box) identified in CRYAB^39^. This region affects the structure and dynamics of the NTD with consequences for CRYAB chaperone activity (^39^; Fig. 2A, purple box). The alignment places the predicted α-helical insertions in HSP20–3 and HSP20–6 (Fig. 2A, B) in this region of the equivalent CRYAB critical-sequence (Fig. 2A). Similar predictions have been made for other sHSPs^67^ and resolved in structural studies e.g.^68^. For CRYAB ssNMR studies reported an α-helix spanning residues 23–32 in CRYAB immediately preceding its critical-sequence^38^, ^39^. The α-helix in the NTD of CRYAB will not fit the ACD central groove, implying that this part of the NTD will likely have to alter its conformation in the dimer^39^.

**Fig. 3:**
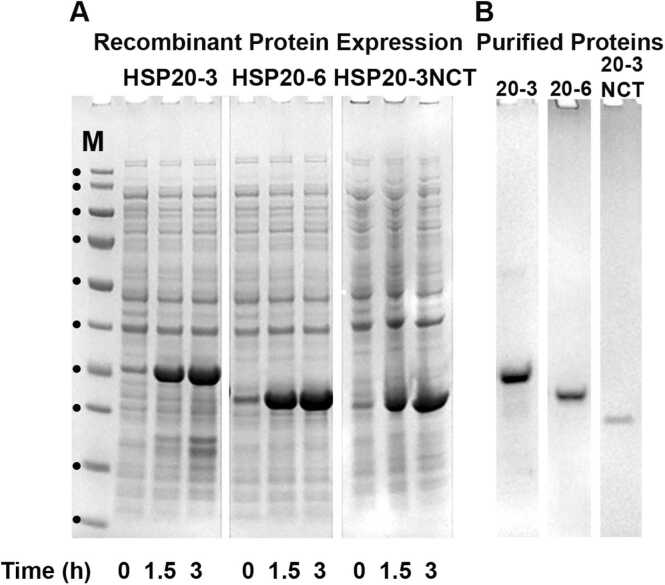
Recombinant expression and purification of HSP20–3, HSP20–6 and HSP20–3NCT. (A) A 3 h time course of the expression of recombinant HSP20–3, HSP20–6 and HSP20–3NCT in *E.coli* after the addition of 1 mM IPTG. (B) Purified HSP20–3, HSP20–6, HSP20–3NCT and human CRYAB after ion exchange and size exclusion chromatography. Protein samples are analysed on 4–12% (w/v) gels using the Bis-Tris buffer system. Molecular weight standards (M) are indicated (•) and in order of increasing electrophoretic mobility correspond to 180, 130, 100, 70, 55, 40, 35, 25, 15 and 10 kDa of the PageRuler™ prestained protein ladder.

Suffice to say, that the sequence differences in the NTD between HSP20–3, HSP20–6 and CRYAB occur in a region with significant influence upon oligomerisation^69^. In the ACD itself, the R120 residue in CRYAB is conserved in HSP20–3 (Fig. 2A, asterisk), but not in HSP20–6. Both HSP20–3 and HSP20–6 have the C-terminal “IPI/V” motif (Fig. 2A, purple bracket) at the end of the ACD, a motif that is important to protein oligomerisation^70^, ^71^. An interaction between the C-terminal domain (CTD; Fig. 2B) of one subunit within the oligomer with the ACD domain of an adjacent subunit^72^, ^73^ is important to oligomerisation. Clearly the five repeats of the QNTN-motif at the very C-terminus of HSP20–3 is striking as there is no equivalent in either CRYAB or HSP20–6 (Fig. 2A, lowercase bold). The function of the QNTN-motif in HSP20–3 with regard to oligomerisation and chaperone activity is clearly an important question, and this was the reason for producing HSP20–3NCT. We therefore proposed to introduce a stop codon immediately after G191 in HSP20–3 (Fig. 2A, arrow) to produce a CTD-truncated form of the sHSP.

### Characterisation of the recombinantly produced HSP20-3, HSP20-6 and HSP20-3NCT

We investigated the oligomerisation properties and chaperone activities of *R. varieornatus* HSP20–6, HSP20–3 and the C-terminally truncated HSP20–3 (HSP20–3NCT), by producing the proteins recombinantly in *E.coli*. The proteins were purified to homogeneity by a combination of ion exchange and size exclusion chromatography. Recombinant human CRYAB was also produced using a similar approach^45^ to provide a comparison and reference point for the observed oligomerisation and chaperone activities of the tardigrade sHSPs.

### Oligomerisation and filament-like features of HSP20-3

**Fig. 4:**
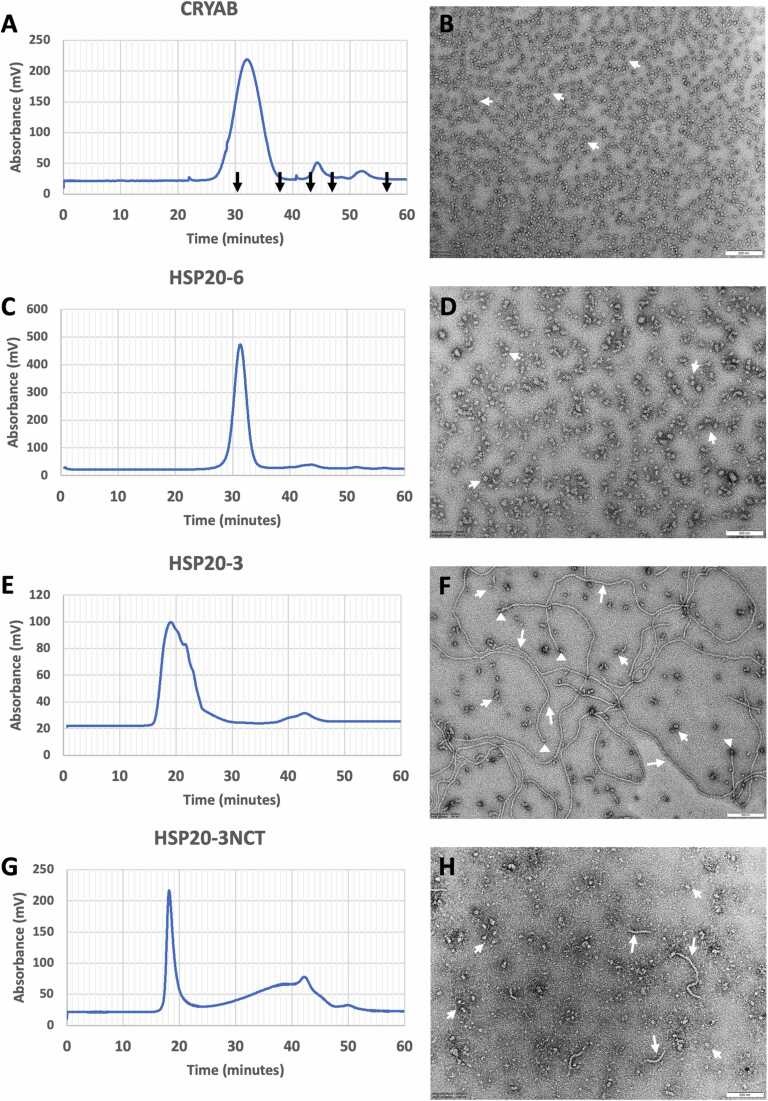
Characterisation of the oligomerisation properties of HSP20–3 and HSP20–6 by SEC and transmission electron microscopy. Recombinant human CRYAB eluted as a symmetrical peak with a maximum at 33 min (A) The elution time of the molecular weight standards, thyroglobulin (670 kDa), γ-globulin (150 kDa), ovalbumin (43 kDa) and ribonuclease (13.7 kDa) and p-aminobenzoic acid (0.137 kDa) are indicated by the arrows. This peak contained oligomeric particles (arrowheads) as revealed by electron microscopy and negative staining of the sample with uranyl acetate (B). By contrast both HSP20–6 (C), HSP20–3 (E) and HSP20–3NCT (G) all eluted earlier from the column. A negatively stained HSP20–6 sample (D; arrowheads) revealed particles similar to CRYAB (B; arrowheads) only larger. For HSP20–3 (F) and HSP20–3NCT (H) “filament-like” structures (arrows) as well as particles (arrowheads) are seen. Some particles locate to the ends of the “filament-like” structures (F, triangles). The “filament-like” structures formed by HSP20–3NCT (H, arrows) were shorter than those in the HSP20–3 sample. The HSP20–3NCT particles are also less uniform in shape (H, arrowheads). Scale bars – 200 nm.

Size exclusion chromatography confirmed that purified HSP20–3, HSP20–3 and HSP20–3NCT all formed oligomers as observed also for recombinant human CRYAB (Fig. 4). HSP20–6 (Fig. 4C) was similar in its peak elution time (31 min) compared to CRYAB (Fig. 4A; 33 min) but both HSP20–3 and HSP20–3NCT eluted much earlier indicating larger oligomers (Fig. 4E, G). These were visualised by negatively staining samples of the three proteins and comparing the structures seen to those formed by recombinant human CRYAB (Fig. 4B). The elution time of the HSP20–6 peak was around 31 min, earlier than CRYAB (33 min). As shown by negatively staining samples and viewing them by TEM, the oligomers formed by HSP20–6 were similar (Fig. 4D) to those seen for CRYAB (Fig. 4B) but noticeably larger than those formed by CRYAB (Fig. 4B) as suggested by the earlier elution time by SEC (Fig. 4A, C).

Both HSP20–3 (Fig. 4E) and HSP20–3NCT (Fig. 4G) eluted much earlier (19 min) than CRYAB (Fig. 4A) suggesting larger oligomeric structures were present than those seen for CRYAB. Electron microscopy of negatively stained samples revealed the presence of both particles (Fig. 4F, H; arrowheads) and filament-like structures in the HSP20–3 (Fig. 4F, arrows) and HSP20–3NCT (Fig. 4H, arrows) samples. The filament-like structures formed by HSP20–3NCT appeared shorter and less abundant than those seen for the full length HSP20–3 (cf Fig. 4F and H). These images also suggested that the particle and filament-like structures seen for HSP20–3 could be interrelated given the close proximity of some particles to filament ends (Fig. 4F; triangles).

**Fig. 5:**
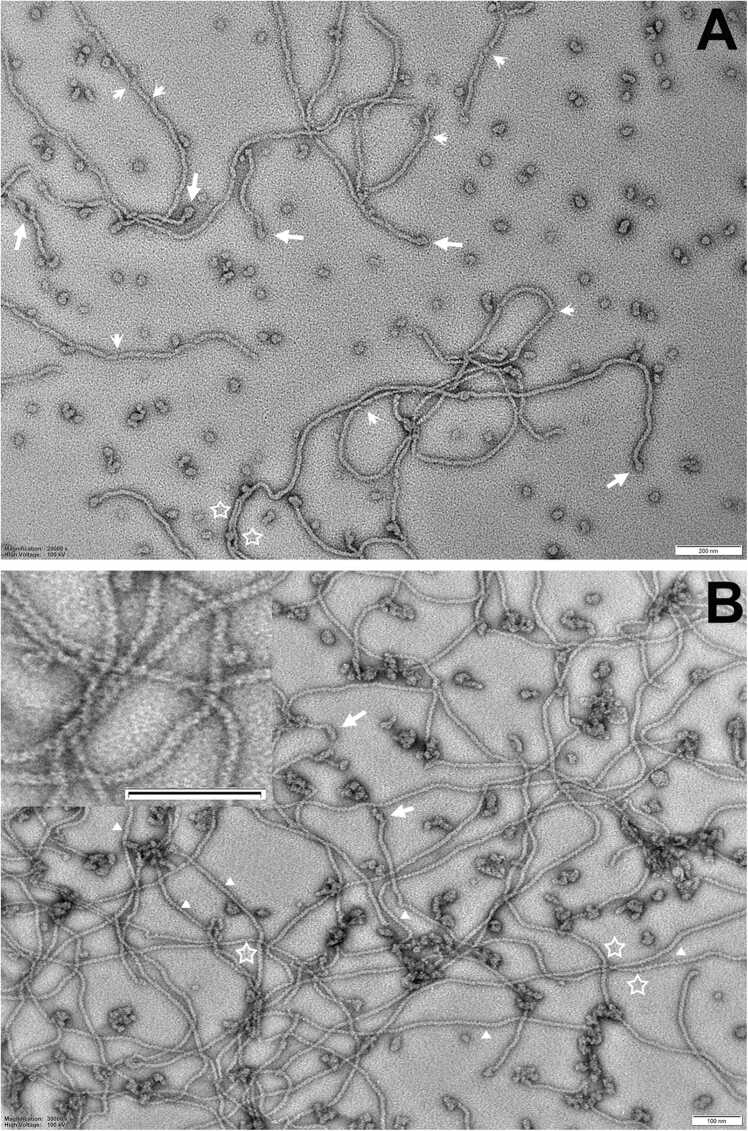
TEM images of negatively stained HSP20–3 to illustrate the assembly landscape and their inter-relationships. In (A), HSP20–3 has both filamentous and particle assemblies and here we present examples showing that at the end of the filaments there are often particles (arrows). The filaments also present with schisms (arrowheads) and the filament width varies, sometimes bifurcating (stars). These images suggest a degree of assembly plasticity previously unseen for other eucaryote sHSPs. In (B), there are further examples of the filament-particle relations (arrows) and variation in filament width (stars), but in addition the filaments are not smooth-walled, but undulating suggesting a periodicity along the length of the filament-like structures (insert). Bars are 200 nm (A) and 100 nm (B) respectively.

Fig. 5 shows a more detailed investigation of the filament ends in the HSP20–3 sample and the relationship to the particles and their elongated intermediates (Fig. 5A, B; white arrows). HSP20–3 filament-like structures are susceptible to localised schisms (Fig. 5A, B; arrowheads), they sometimes appear bifurcated and the filament width varies from 4 to 12.5 nm (Fig. 5A, B; stars). At higher magnification (Fig. 5B; insert) the surface of the filament-like structure does not appear smooth, rather it appears to have a periodicity. The particles are equally variable in aspect ratio, width and shape indicative of a varied assembly landscape for HSP20–3. Interestingly, removal of the C-terminal repeat sequences from HSP20–3 did not prevent the formation of the filament-like structures (Fig. 4H), but the filament-like structures appeared shorter than those observed for the full length HSP20–3 (Fig. 4F).

### Chaperone activities of HSP20-3 and HSP20-6 compared to human CRYAB

**Fig. 6:**
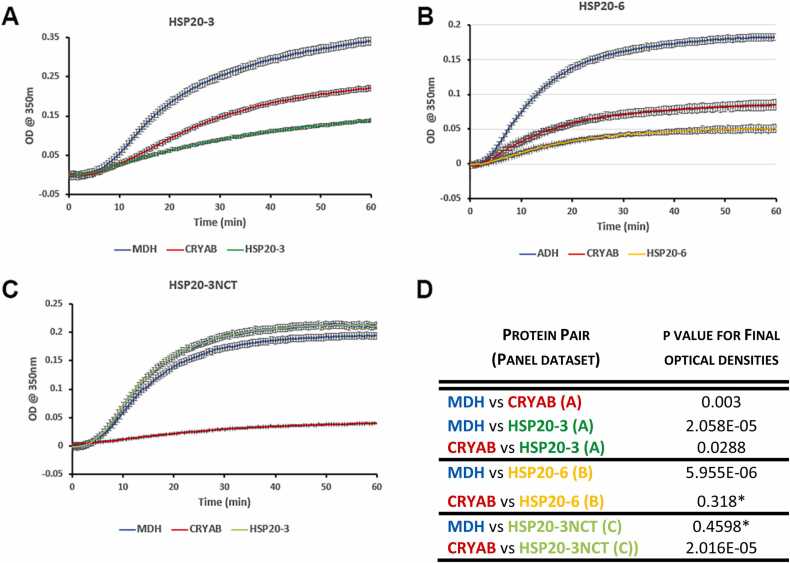
Chaperone assay data for HSP20–3, HSP20–6 and HSP20–3NCT using the client protein MDH. The ability of HSP20–3 (A), HSP20–6 (B) and HSP20–3NCT (C) to inhibit the temperature induced (45 ^0^C) aggregation of malate dehydrogenase (MDH) was monitored by following the change in absorbance at 350 nm for 60 min. The change in absorbance for MDH alone (blue line) as well as in the presence of CRYAB at a 1:4 molar ratio to MDH (red line) are included as controls. The HSP20–3 (A, dark green line) and HSP20–3NCT (C; light green line) are also added in a 1:4 molar ratio to MDH and the change in absorbance recorded. Error bars are standard errors of the mean. (D) Statistical analysis of the MDH and sHSP combinations. The calculated p values for protein pairs from the identified datasets are shown. Asterisks indicate those pairs for which no significance was observed.

To assess the chaperone potential of *R.varieornatus* HSP20–3 and HSP20–6 to protect against heat induced aggregation we selected MDH as a client protein^5^ and compared their activity to that of recombinant human CRYAB (Fig. 6). Both tardigrade proteins and CRYAB were able to suppress the heat-induced aggregation of MDH evidencing that both HSP20–3 and HSP20–6 had chaperone activity (Fig. 6A, B, D). HSP20–3 (Fig. 6A) was significantly better than CRYAB in this MDH client protein assay (Fig. 6D). HSP20–3 is the most highly expressed sHSP induced by heat shock in *R. varieornatus* (Fig. 1) and these data suggest it is an efficient chaperone in this particular client protein assay, although more detailed studies of these and the other sHSPs expressed in *R. varieornatus* are needed to assess their chaperone profiles aas has been done for human sHSPs^5^. Removal of the QNTN-motif repeat from the very C-terminus of HSP20–3 abolished the chaperone activity of HSP20–3 (Fig. 6C, D).

## Discussion

### HSP20-3 and HSP20-6 are bone fide sHSP chaperones induced by heat shock in *R.varieornatus*

A 35 ^0^C heat shock very significantly upregulates the transcription of two sHSPs in *R.varieornatus*, namely HSP20–3 and HSP20–6 (Fig. 1A). In fact HSP20–3 is one of the most upregulated genes in this animal after the heat shock (Table 2) and rightly deserves the term “inducible”. It is usually the HSP70 class of protein chaperones that are tmhe most transcriptionally upregulated after stress^74^; as such the 8.62 log2 fold change in HSP20–3 transcripts (Table 2) is expected to be functionally important. Comparison of the heat shock induced changes in the expression of the other members of the sHSP gene family in *R.varieornatus*, revealed there is a range of different responses. These varied from little change (HSP20–2, HSP20–8) and even reduction (HSP20–7) to a several fold increase (HSP20–1, HSP20–4, HSP20–5), suggesting a more complex regulation indicative perhaps of diverse functions for the various sHSPs in *R.varieornatus.* With a complement of 8 sHSPs^7^, *R. varieornatus* is not dissimilar to mammals that express 10 sHSPs^75^, ^76^. The structure, assembly, dynamics and function of mammalian sHSPs are diverse^22^, ^24^, ^36^, ^69^, ^74^. For example CRYAB (HSPB5) forms polydisperse multi-subunit oligomers^73^, ^77^, ^78^ and binds a wide spectrum of client proteins^18^, ^79^, ^80^, ^81^, whilst HSPB6 forms only dimers and demonstrates poor chaperone activity^82^, ^83^. HSPB3 is developmentally regulated involved with muscle cell differentiation^84^ and it forms hetero-tetramers with HSPB2^85^. In the nematode, *Caenorhabditis elegans*, there are 16 sHSPs expressed^7^, ^86^ with similar variability in their structure-function properties^87^. The HSP-12 class of sHSPs within *C.elegans* are critical for dauer formation, longevity and fecundity^88^, but these proteins do not oligomerise and show poor *in vitro* chaperone activity^88^, ^89^. By contrast, most of the HSP-16 class of sHSPs, do oligomerise and have classical chaperone properties shown by *in vitro* chaperone assays^87^, ^90^. There are exceptions, such as HSP-17, that is reported to function as a selective aggregase^91^. Nevertheless, depletion of HSP-17 negatively affects heat shock survival, fecundity and lifespan^91^ indicating that sHSP function is complex. It is important to investigate each sHSP independently to establish its own specific structure-function profile, as we have shown here for HSP20–3 and HSP20–6. Indeed both of these sHSPs are also amongst the most highly expressed in the tun state (Supplementary Fig. 2) and it is reasonable to expect some similarities but also differences in the function of these two sHSPs in mitigating heat shock as well as anhydrobiosis. The tardigrade *H. exemplaris* expresses 9 sHSPs^41^ and a recent study on its desiccation response identified HSP24.6 as particularly good at promoting desiccation survival in a bacterial model and it was a better chaperone in heat-induced aggregation of client proteins than HSP21^41^. Both of these sHSPs from *H. exemplaris* form large oligomeric complexes of variable size, but neither were found to form filament-like structures and none of the sHSP complement in *H. exemplaris* possessed the QNTN-repeat sequence in a CTD as seen for HSP20–3 (Fig. 2A).

### The structure-function properties of *R.varieornatus* HSP20-3 and HSP20-6

Here, for the first time, we have assessed the chaperone and oligomerisation potential of HSP20–3 and HSP20–6 from *R.varieornatus*. Both were efficient chaperones as compared with human CRYAB (Fig. 6A, B, D) in a client protein heat aggregation assay using MDH. HSP20–3 significantly better than CRYAB as a chaperone in this assay (Fig. 6D). Future studies are needed to characterise in detail their chaperone properties in the context of the sHSP complement in *R.varieornatus*^5^. The most remarkable finding, however, was the formation of the filament-like structures by HSP20–3 (Fig. 4F and Fig. 5A, B). The five-fold repeat of the QNTN-motif at the end of the CTD was clearly important to both the chaperone and oligomerisation potential for HSP20–3 as removal of these sequences, produced a protein, HSP20-3NCT, that had lost its chaperone activity and reduced its filament-forming potential (Fig. 4H and Fig. 6C). A recent study investigating two sHSPs from the tardigrade *H. exemplaris*, HSP21 and HSP24.6^41^ found both formed oligomers and protected a client protein against heat induced aggregation, although HSP24.6 was the better of the two sHSPs^41^. Neither HSP21 or HSP24.6 formed oligomeric structures resembling filament-like structures, but then none of the *H. exemplaris* sHSPs possess a repeat-motif in their CTD. Phylogenetic comparison of the *H. exemplaris* and *R.varieornatus* sHSPs (Supplementary Fig. 3) suggest that HSP24.6 and HSP20–6 are homologues, whilst HSP20–3 though part of the HSP21 clade, is distinctive by the presence of the repeated QNTN-motif that form a large intrinsically disordered region in the CTD (Supplementary Fig. 3) as predicted by AlphaFold2 (Fig. 2B).

Another interesting feature of the two tardigrade sHSPs is the insertion (Fig. 2A, purple box) within their NTDs. The insertion is adjacent to the CRYAB critical-sequence (23−32) in the middle domain of the NTD as judged by their homology to CRYAB (Fig. 2A, rectangle). The critical-sequence is important for subunit-subunit interactions and therefore for both chaperone activity and oligomerisation^39^. The archael sHSP, HSP16.5, can be engineered to form an array of oligomeric forms from 30 to 38 subunits by the insertion of a proximal NTD sequence (57−72) from HSP27 (HSPB1) at the junction between the NCD and ACD to affect oligomer size^92^. Insertions into the NTD around the critical-sequence can therefore influence the oligomerisation and this also appears to be the case for both HSP20–3 and HSP20–6. AlphaFold2 predicts that the insertions in *R.varieornatus* sHSPs will be α-helical, albeit with low confidence (Fig. 2B), and it is striking that in Mj16.5 there is also an AlphaFold2 predicted α-helix^93^ immediately preceding the equivalent critical-sequence in the NTD, suggesting that this is a NTD feature in these two sHSPs worthy of future attention.

### The potential role(s) for the filament-like property of HSP20-3 in *R.varieornatus*

Here we report the first sHSP, HSP20–3, with the ability to form filament-like structures without the need for either aging-mediated modifications^94^ or denaturants and heat, which is the case for CRYAB^42^. These filament-like structures appear directly related to the oligomeric particles also seen in HSP20–3 samples. Based on the observation that these oligomeric particles can be present specifically at the ends of the filaments, we suggest the possibility that they nucleate their assembly, and/or that the filament is constituted from re-arranged particle subunits (Fig. 5A, B). The filament-like structures are not smooth-walled likely due to subunit polymerisation as evidenced by the periodicity along the filament (Fig. 5B). The filament-form of CRYAB remains an active chaperone^42^ as does HSP20–3 (Fig. 6B) so these diverse oligomeric forms are compatible with its chaperone function. Our data show that the repeated QNTN-motif in the CTD is important to the chaperone activity of HSP20–3 (Fig. 6C), however, its removal does not completely abolish filament formation (Fig. 4H). A key question then is what is the function of these filament-like structures formed by HSP20–3 and what advantage does it afford to *R.varieornatus* and its heat stress response?

We suggest that this could be related to protein condensate formation and the protection this affords during heat shock. For CRYAB, it is the spectrum of oligomeric forms and the ability to form elongated polymorphic structures that is important to its function^43^ and we have shown here that for HSP20–3 the spectrum includes filamentous structures (Fig. 5A, B). These filaments will facilitate oligomerisation and increase multivalency, which are key aspects of condensate formation as seen for TDP-43 in the disease amylotrophic lateral sclerosis^95^. Proteins with intrinsically disordered regions are critical to desiccation survival^11^ and tardigrades express a unique group of proteins to facilitate this, namely the abundant heat soluble proteins CAHS, SAHS^6^ and MAHS^8^. The condensate-forming properties of the CAHS proteins also involves their ability to assemble into a filament-like network, a vital structural transition needed to support phase separation as part of the desiccation tolerance of tardigrades^14^. Such condensates and vitrification are essential to anhydrobiosis, but also to the high-temperature tolerance^96^. Whilst CAHS proteins are restricted to *Eutardigrada*^97^, the biological principle of coupling filamentous polymers with protein stabilisers to facilitate condensate formation is conserved across the plant and animal kingdoms^14^, ^16^, ^43^, ^98^, ^99^. This is no more so than in the eye lens, where temperature-induced phase separation of the crystallins depends upon the presence of intermediate filaments^100^. Indeed CRYAB co-polymerises with the two lens specific intermediate filaments, BFSP1 and BFSP2, to form beaded filaments^101^. Intermediate filaments and CRYAB are binding partners^18^, ^79^, and both CRYAB^39^ and intermediate filaments^102^ possess intrinsically disordered domains that support their phase separation under the appropriate conditions. The most highly induced heat shock sHSP chaperone HSP20–3 has combined filaments and particles with its chaperone function providing a far wider range of polymeric structures than HSP20–6, which could be the reason why this is the most highly induced sHSP in response to heat stress for *R.varieornatus*.

## Declaration of Competing interest

No competing interests.
