# Supplementary material for: The major inducible small heat shock protein HSP20-3 in the tardigrade Ramazzottius varieornatus forms filament-like structures and is an active chaperone
Source: Cell Stress Chaperones. 2023 Dec 5;29(1):51–65. doi: 10.1016/j.cstres.2023.12.001 (PMC10939073; doi:10.1016/j.cstres.2023.12.001)

# Supplementary Data

Figure 1: LC-MS analysis of recombinant expressed and purified proteins

The primary sequence and the identified peptides (blue lines) are indicated for each of the recombinant proteins used in the study.


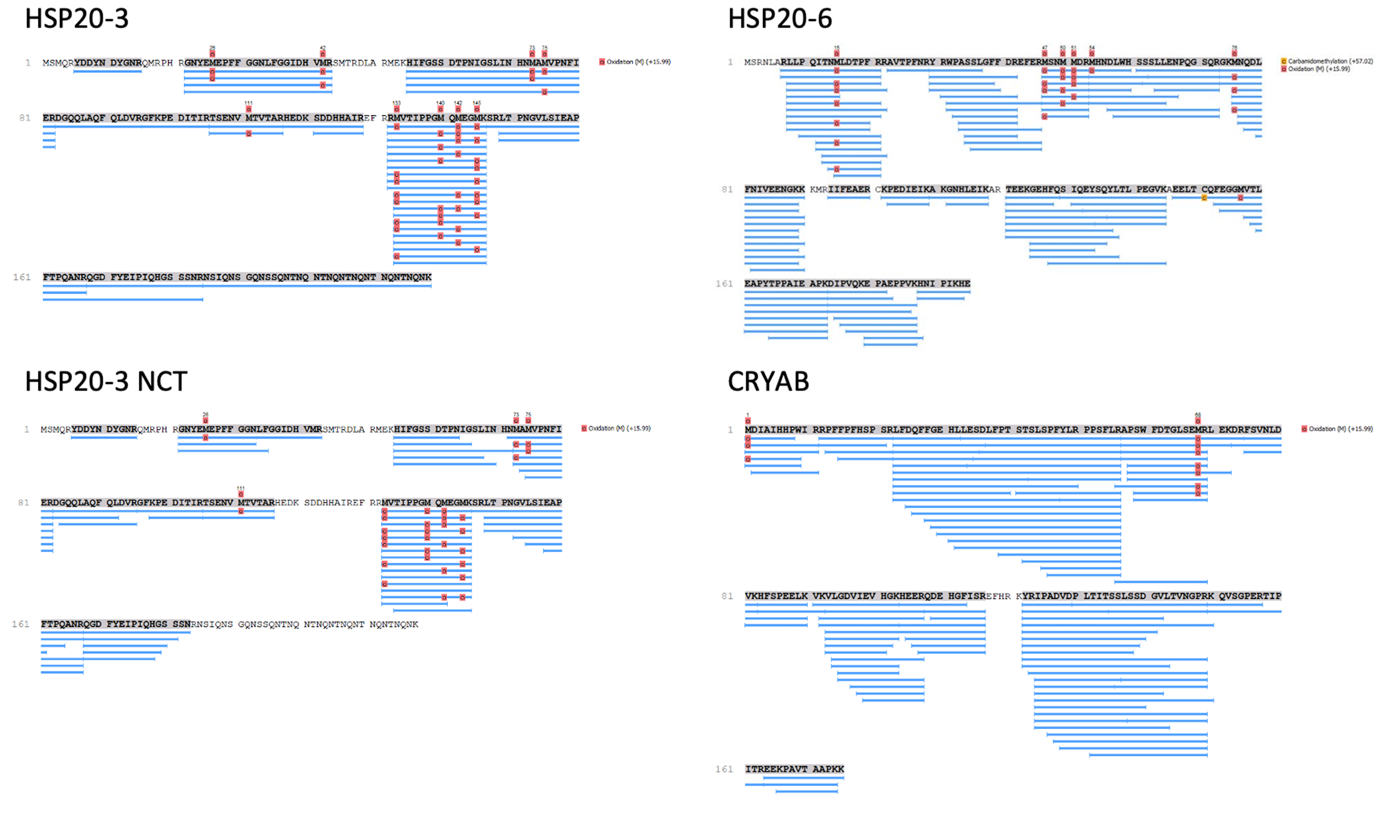


### Figure 2: Expression (log2 normalised counts) of small heat shock genes in R.varieornatus

*at different developmental stages and states available under the ENA accession numbers PRJNA369262 and PRJNA533981.* Each group has three replicates (white circles). Mean values (black dots) and the corresponding ±standard error (solid line) are shown.

###
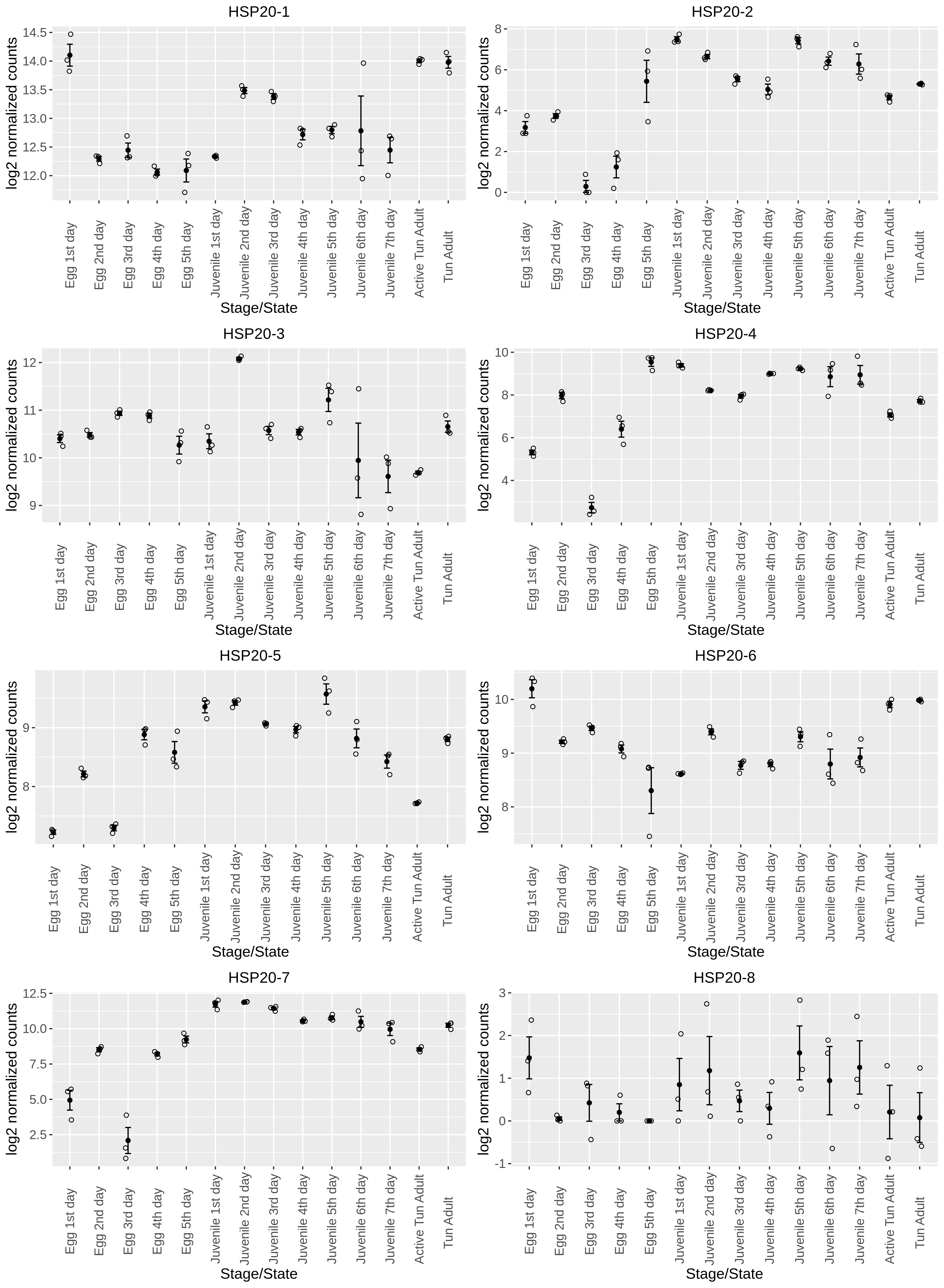


## Figure 3. Gene tree with standard bootstrap and alignment of the H. exemplaris and R.varieornatus sHSPs.

## The gene tree was generated using msa v1.32.0 (https://bioconductor.org/packages/release/bioc/html/msa.html) and iqtree2 v2.2.3 (<https://github.com/iqtree/iqtree2>) with 1000 bootstrap and rooted using two yeast sHSPs (Sc HSP26 and Sc HSP42). The protein alignment was generated using Clustal Omega (<https://www.ebi.ac.uk/Tools/msa/clustalo/>). Notice the relationship between HSP24.6 and HSP20-6 and the relationship of HSP20-3 to H. exemplaris HSP21, HSP17, HSP19 and HSP20 and to R. varieornatus HSP20-1. The protein sequence alignment of all sHSPs identified in H. exemplaris and R. varieornatus is also shown.


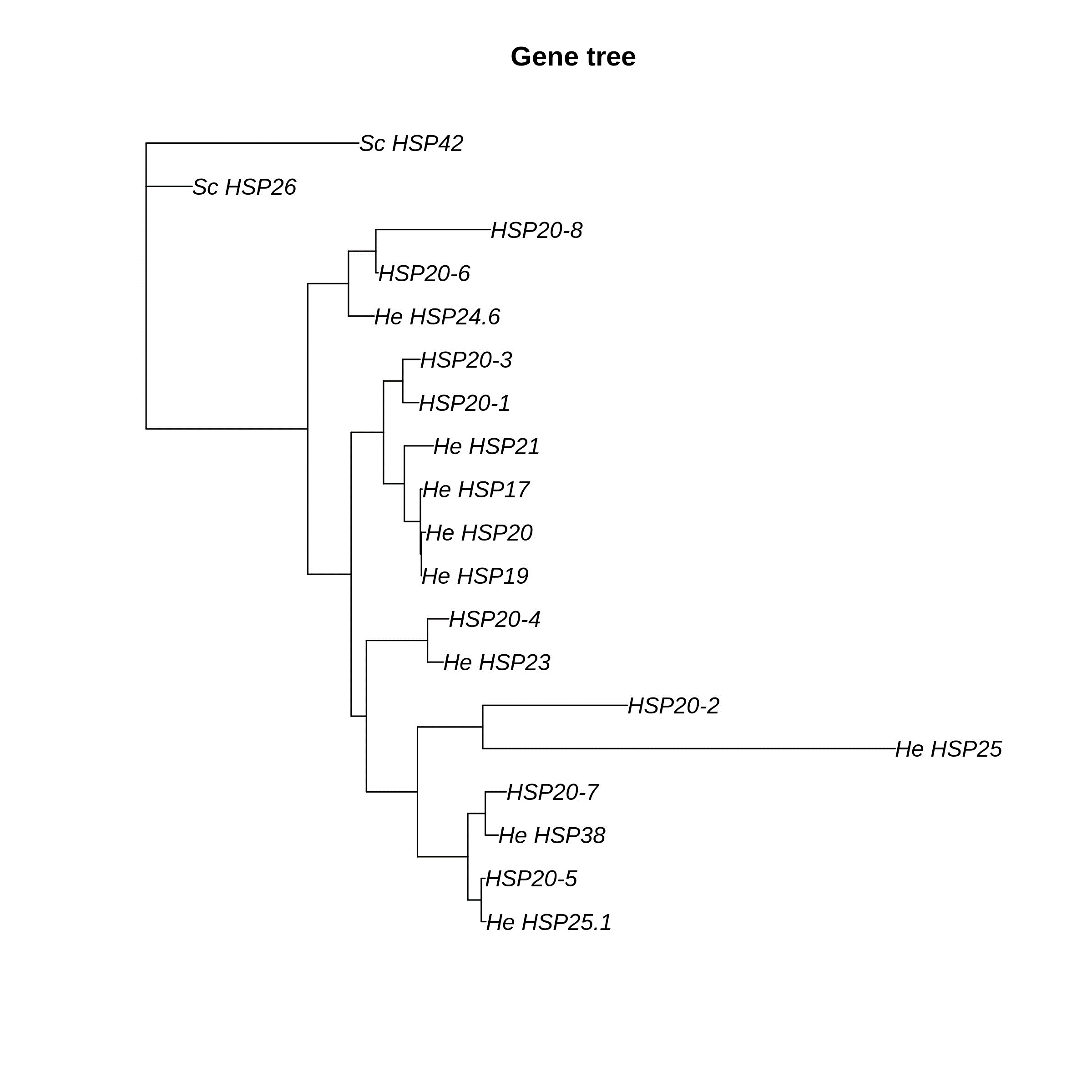


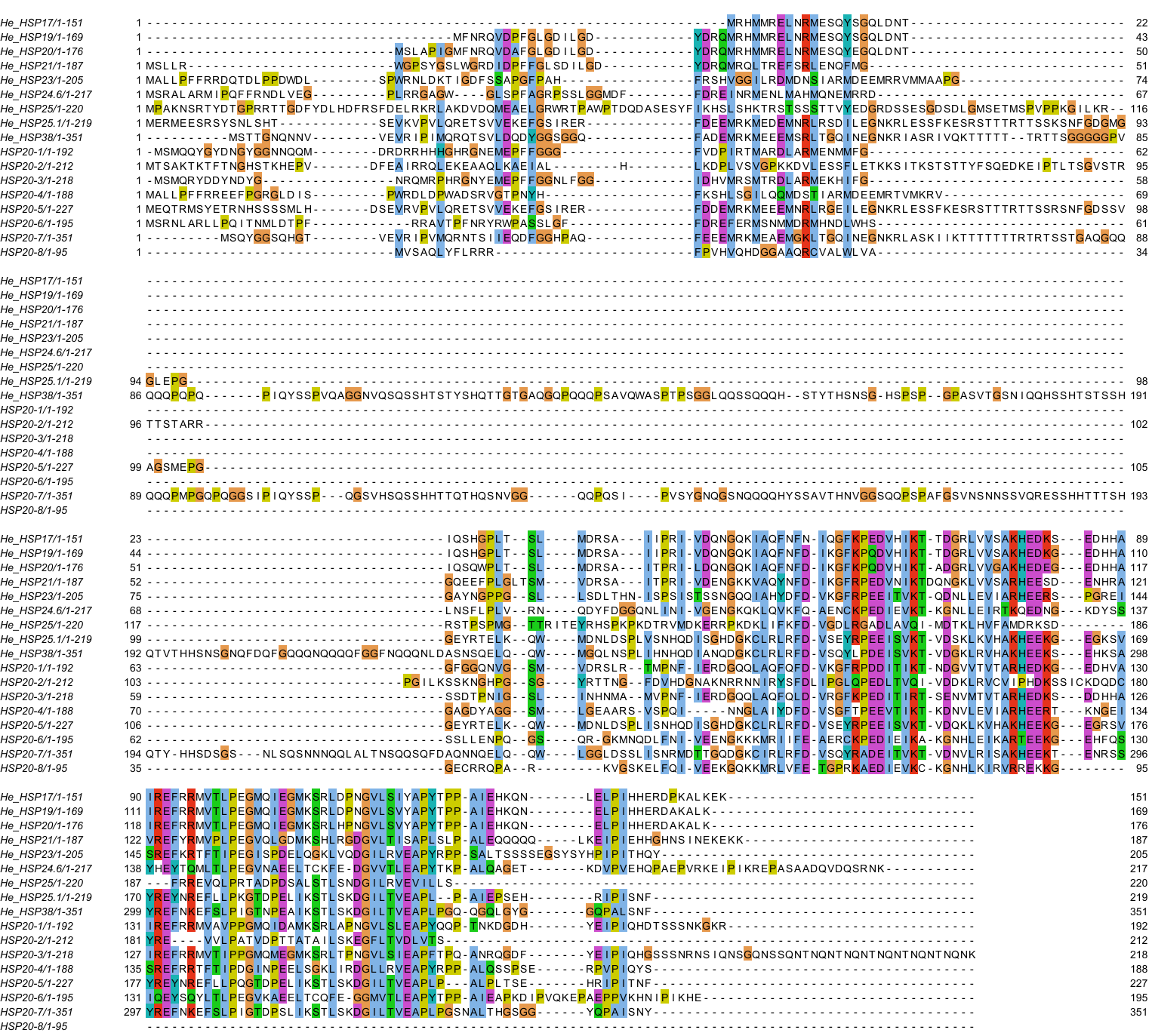

Supplement: Supplementary file 1 — Supplementary material [file mmc1.docx]
